# Mimicking the Neurotrophic Factor Profile of Embryonic Spinal Cord Controls the Differentiation Potential of Spinal Progenitors into Neuronal Cells

**DOI:** 10.1371/journal.pone.0020717

**Published:** 2011-06-17

**Authors:** Masaya Nakamura, Osahiko Tsuji, Barbara S. Bregman, Yoshiaki Toyama, Hideyuki Okano

**Affiliations:** 1 Department of Orthopaedic Surgery, Keio University, Tokyo, Japan; 2 Department of Neuroscience, Georgetown University Medical Center, Washington, D. C., United States of America; 3 Department of Physiology, Keio University, Tokyo, Japan; University of South Florida, United States of America

## Abstract

Recent studies have indicated that the choice of lineage of neural progenitor cells is determined, at least in part, by environmental factors, such as neurotrophic factors. Despite extensive studies using *exogenous* neurotrophic factors, the effect of *endogenous* neurotrophic factors on the differentiation of progenitor cells remains obscure. Here we show that embryonic spinal cord derived-progenitor cells express both ciliary neurotrophic factor (CNTF) and brain-derived neurotrophic factor (BDNF) mRNA before differentiation. BDNF gene expression significantly decreases with their differentiation into the specific lineage, whereas CNTF gene expression significantly increases. The temporal pattern of neurotrophic factor gene expression in progenitor cells is similar to that of the spinal cord during postnatal development. Approximately 50% of spinal progenitor cells differentiated into astrocytes. To determine the effect of endogenous CNTF on their differentiation, we neutralized endogenous CNTF by administration of its polyclonal antibody. Neutralization of endogenous CNTF inhibited the differentiation of progenitor cells into astrocytes, but did not affect the numbers of neurons or oligodendrocytes. Furthermore, to mimic the profile of neurotrophic factors in the spinal cord during embryonic development, we applied BDNF or neurotrophin (NT)-3 exogenously in combination with the anti-CNTF antibody. The exogenous application of BDNF or NT-3 promoted the differentiation of these cells into neurons or oligodendrocytes, respectively. These findings suggest that *endogenous* CNTF and *exogenous* BDNF and NT-3 play roles in the differentiation of embryonic spinal cord derived progenitor cells into astrocytes, neurons and oligodendrocytes, respectively.

## Introduction

Neural stem/progenitor cells are an ideal source of tissue for neural transplantation, since these cells can be expanded in vitro, maintained in an undifferentiated state and retain the capacity to differentiate into neurons, astrocytes and oligodendrocytes [1], [2], [3]. Previous in vitro studies, however, showed that when neural progenitor cells were permitted to differentiate, they gave rise to mainly glial cells and only smaller fraction developed into neurons [4], [5], [6], [7], [8]. Although the culture systems used by various authors have differed with regard to the species, anatomic location, and developmental age of the harvested tissue, there is growing evidence to suggest that the choice of lineage is determined, at least in part, by environmental factors, among which neurotrophic factors play a potential modulating role in differentiation of progenitor cells [2], [9], [10]. For example, the *exogenous* addition of ciliary neurotrophic factor (CNTF) and subsequent gp130-signal activation promotes the differentiation of astrocytes from embryonic hippocampus, cortex and spinal cord derived progenitor cells [11], [12], [13], [14], [15], [16]. Brain-derived neurotrophic factor (BDNF) induces neuronal differentiation from embryonic striatum, subependymal zone and hippocampus derived progenitor cells [4], [9], [17], [18]. Neurotrophin-3 (NT-3) induces neuronal differentiation from embryonic cortex and hippocampus derived progenitor cells [18], [19]. Despite these extensive studies using the *exogenous* application of neurotrophic factors, little is known about the effect of *endogenous* neurotrophic factors on the differentiation of progenitor cells. We suggest that differentiation of neural progenitor cells is regulated not only by *exogenous* level of growth factors but rather, the *endogenous* expression of growth factors also play crucial roles in the differentiation of neural progenitor cells. In this study, we sought to determine the extent to which differentiation of spinal progenitor cells is altered by manipulation of *endogenous* as well as *exogenous* neurotrophic factors in vitro. We focused on the spinal progenitor cells because spinal progenitor cells may be distinct from cortical progenitor cells [20]. A better understanding of the control mechanism of spinal progenitor cell's fate should enhance efforts to develop effective transplantation strategies aimed at restoring functional connectivity in the injured spinal cord.

In the present study, we used ribonuclease (RNase) protection assay to detect the expression of multiple neurotrophic factors genes in both developing spinal cord and embryonic spinal cord derived-progenitor cells. We used triple epitope immunocytochemistry to determine the phenotypic fate of progenitor cells in vitro under different treatments, including neutralization of endogenous CNTF and/or administration of exogenous BDNF or NT-3. The data indicate that *endogenous* CNTF and *exogenous* BDNF and NT-3 play crucial roles in the differentiation of E14 spinal cord derived- progenitor cells into astrocytes, neurons and oligodendrocytes, respectively.

## Results

### Neurotrophic factor gene profile of spinal cord during normal development

The development and maturation of progenitor cells depend not only on their genetic programming, but also on sequences and contributions of various environmental signals such as neurotrophic factors that are appropriate to their developmental stages. How neurotrophic factors precisely control cell fate and orchestrate the generation of neurons, astrocytes and oligodendrocytes in vivo is not clearly understood. To determine how neurotrophic factor gene expression is regulated in the spinal cord during normal development, we examined the neurotrophic factor gene expression profiles of developing spinal cord between E14 and P17 by RNase protection assay.

**Figure 1:**
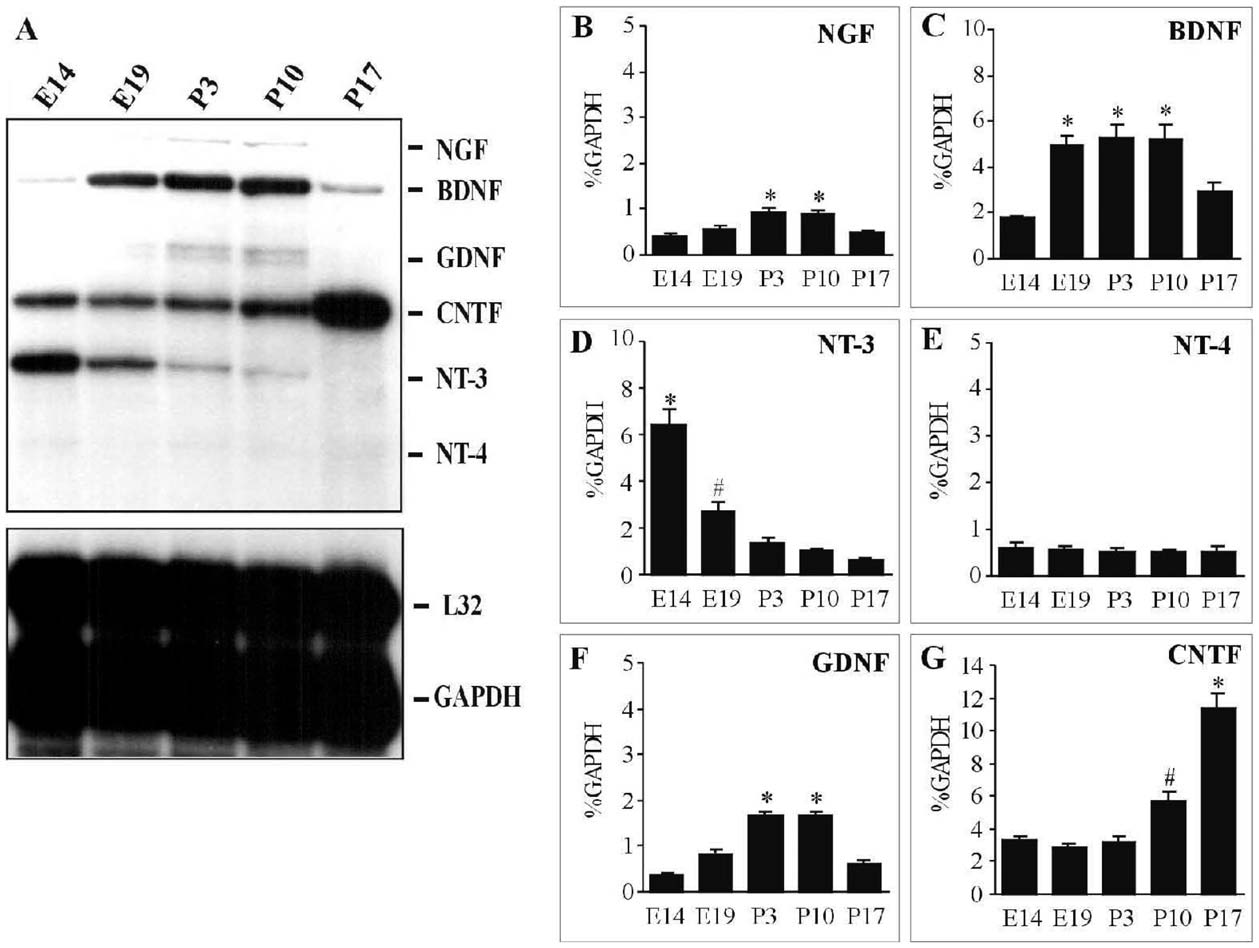
Neurotrophic factor gene profile of spinal cord during development. Spinal cord tissues were harvested from embryos or neonates at each developmental age. Total RNA (10 µg) was loaded at each lane for RNase protection assay (A). There were three different temporal patterns of neurotrophic factor gene expression. NT-3 gene expression peaked at E14 and then declined sharply (D). BDNF gene expression (C), followed by NGF (B) and GDNF (F), increased between E19 and P10. Initial moderate expression of CNTF increased significantly at P10 and P17 (G). Each value represented mean ± SEM of % GAPDH obtained from four independent experiments. GAPDH was used as an internal control to normalize for loading differences. * Significant difference compared to the other developmental ages except (*), # Significant difference compared to the other developmental ages including (*) (p<0.05, Tukey *post hoc* test).

A high level of NT-3 gene expression, a moderate level of CNTF and a low level of BDNF were observed in E14 spinal cord, but the bands for NGF, GDNF or NT-4 were undetectable (Fig. 1*A*). These neurotrophic factors showed distinct temporal patterns of their gene expression during embryonic and postnatal development. The high level of NT-3 gene expression at E14 decreased sharply by birth and subsequently there was almost no expression of NT-3 at P17 (Fig. 1*D*). BDNF gene expression, which was low at E14, increased between E19 and P10 (Fig. 1*C*). Following the increase of BDNF gene expression, NGF and GDNF gene expression increased (Fig. 1*B, F*). The temporal pattern of NGF and GDNF gene expression was similar to that of BDNF, but the magnitude of increase of NGF and GDNF gene expression was less than that of BDNF. Levels of NGF, BDNF and GDNF gene expression decreased simultaneously between P10 and P17. Consistent with this decrease of BDNF, GDNF and NGF gene expression, CNTF gene expression increased significantly at P17 (Fig. 1*G*).

### Neurotrophic factor gene profiles of spinal progenitor cells during differentiation

**Figure 2:**
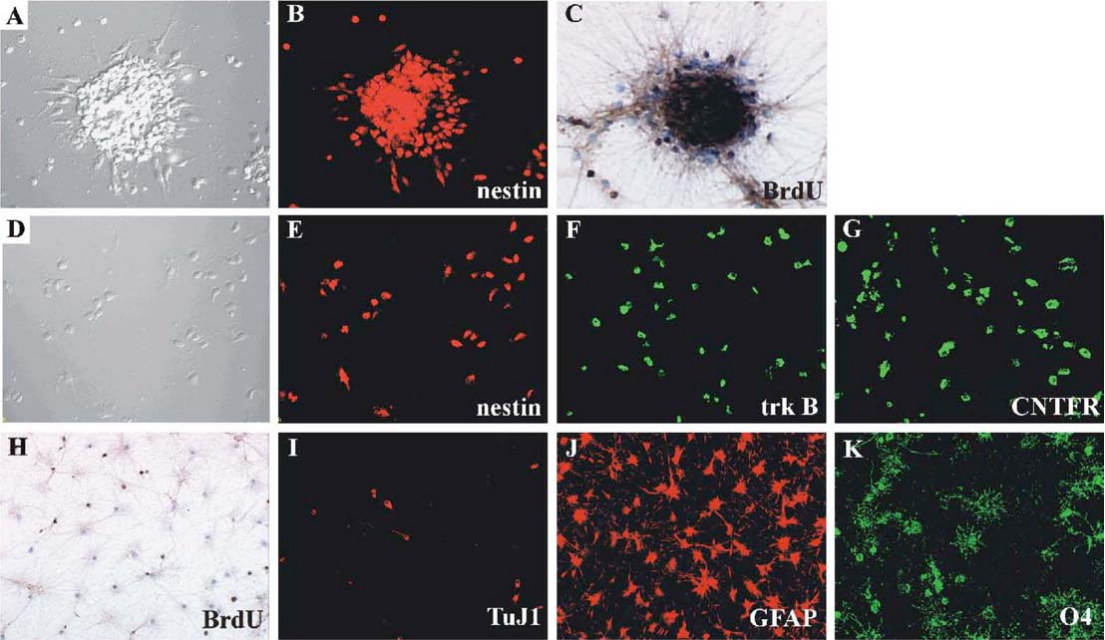
Characterization of E14 spinal cord-derived progenitor cells. Progenitor cells obtained from E14 fetal spinal cord formed sphere in the growth medium containing EGF/FGF2 (A: phase contrast image) and they were nestin (B: identical field to A) and BrdU immunoreactive (C). After dissociation, these cells were seeded on PEI-coated coverslips in no EGF/FGF2 and 1%FBS containing culture medium (D: phase contrast image). At 1div, over 90% of the dissociated progenitor cells were still nestin immunoreactive (E: identical field to D) and there were also trkB (F) and/or CNTFRα immunoreactive (G). These cells slowed or stopped dividing and during differentiation about 30% of the cells were labeled with BrdU at 7 div (H). At 7 div, these cells were triple-stained with anti-TuJ1 for neuron (I), GFAP for astrocyte (J) and O4 for oligodendrocyte (K). Approximately 50% of these cells differentiated into astrocytes.

Progenitor cells were isolated from E14 rat spinal cord and mechanically dissociated in culture medium. These cells expanded to form “neurospheres” in the presence of EGF/FGF2 and over 95% of these cells were nestin-immunoreactive (Fig. 2*A, B*). BrdU-labeling of these clusters confirmed that about 90% of the cells proliferated as determined by nuclear BrdU corporation (Fig. 2*C*). At 7 div, these neurospheres were centrifuged and dissociated mechanically. Single cells were subsequently re-seeded into EGF/FGF2-containing medium. This procedure resulted in a second generation of neurospheres. After 2–4 passages, these cells were seeded into the PEI-coated flasks or coverslips with differentiation medium (EGF/FGF2-free and 1%FBS) following dissociation. At 1 div over 90% of these cells were still nestin immunoreactive (Fig. 2*D, E*) and there were also trkB and/or CNTFRα immunoreactive (Fig 2*F, G*). In the differentiation medium, these cells slowed or stopped dividing. At 7 div about 30% of these cells were labeled with BrdU during differentiation (Fig. 2*H*), and about 80% of these cells differentiated into neurons, astrocytes or oligodendrocytes (Fig. 2*I, J, K*). Quantitative analysis revealed that majority of differentiated cells was astrocytes (52%), while fewer cells differentiated into oligodendrocytes (23%) and neurons (4%). This suggests that conditioning of the medium by the differentiating progenitor cells, especially *endogenous* neurotrophic factors may enhance glial cell differentiation.

**Figure 3:**
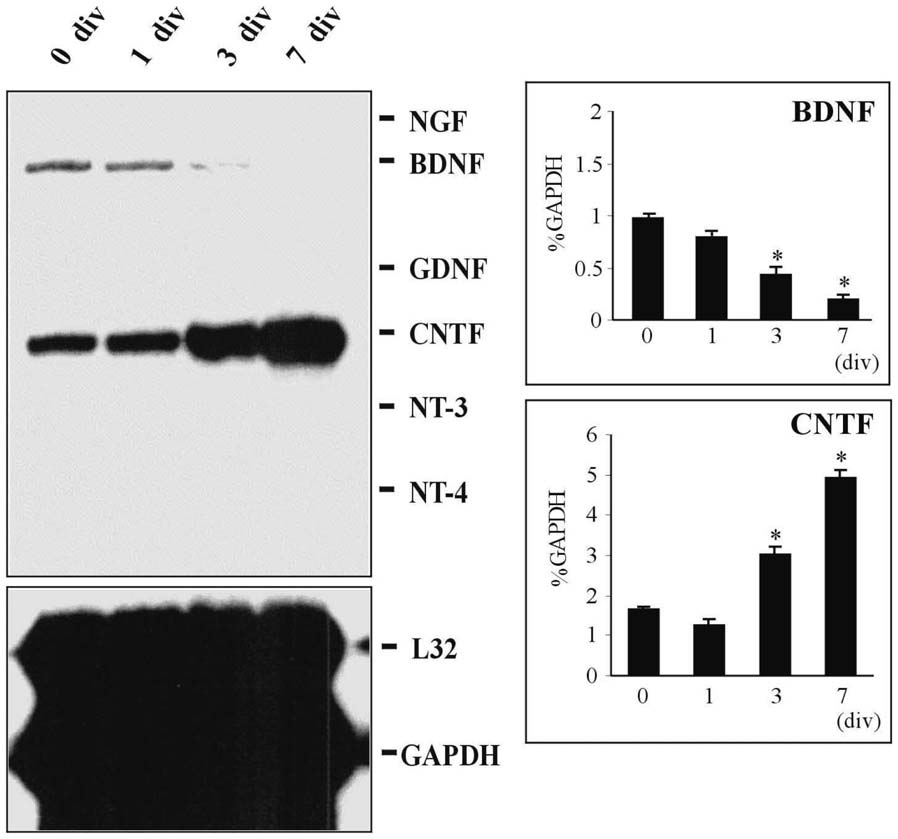
Neurotrophic factor gene profile of E14 spinal cord derived-progenitor cells during differentiation *in vitro.* After 2–4 passages, cells were seeded on the PEI-coated culture flasks at a density of 2×10^5^ cells/ml in the differentiation medium (no EGF/FGF2, 1% FBS). Culture cells were harvested at 1, 3 and 7 days in vitro (div). As a control cells were harvested immediately after dissociation (0 div). Total RNA (5 µg) was loaded at each lane for RNase protection assay. Before differentiation (0 div), there were two prominent gene expression of BDNF and CNTF. While BDNF gene expression decreased significantly at 3 and 7 div, CNTF gene expression increased significantly at 3 and 7 div. Each value represented mean ± SEM of % GAPDH obtained from three independent experiments. GAPDH was used as an internal control to normalize for loading differences. * Significant difference compared to 0 div (p<0.05, Tukey *post hoc* test).

To determine how neurotrophic factor gene expression is regulated during the differentiation of E14 spinal cord derived progenitor cells, we examined the neurotrophic factor gene expression profiles of these cells by multi-probe RNase protection assay. BDNF and CNTF gene expression were observed in spinal progenitor cells before differentiation (0 div) (Fig. 3). During their differentiation BDNF gene expression declined and then became undetectable at 7 div. In contrast, CNTF gene expression increased significantly at 3 div and by 7 div reached the levels 3-fold greater than that of before differentiation (0 div). The expression of NGF, NT-3, NT-4 or GDNF mRNA was undetectable at any time point examined. Thus, E14 spinal cord derived progenitor cells showed a different pattern of neurotrophic factor gene expression during differentiation compared to the normal embryonic developing spinal cord (Fig. 1), including up-regulation of CNTF gene expression, down-regulation of BDNF gene expression and a lack of NT-3 gene expression. Interestingly, this pattern of neurotrophic factor gene expression is similar to that observed during postnatal development, and correlated temporally to active gliogenesis. Furthermore, spinal progenitor cells expressed CNTFR-immunoreactivity during differentiation. Taken together, these findings have led us to propose that CNTF acts as an autocrine and/or paracrine signal involved in the astrocytic differentiation from spinal progenitor cells.

**Figure 4:**
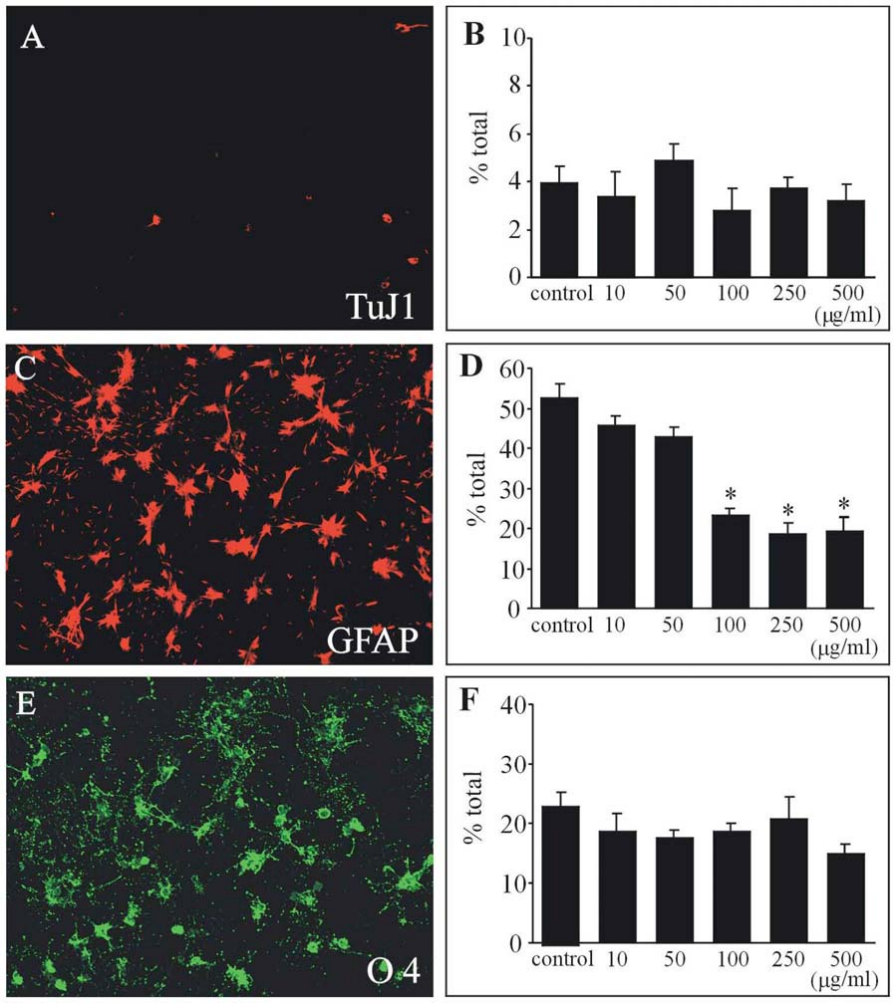
Neutralization of endogenous CNTF inhibited the differentiation of spinal progenitor cells into astrocytes. After 2–4 passages, cells were seeded on PEI-coated coverslips at a density of 2×10^5^ cells/ml in the differentiation medium with different concentrations of anti-CNTF antibody (0–500 µg/ml). At 7div the culture cells were triple-stained with TuJ1 (A), GFAP (C) and O4 (E). Neutralization of endogenous CNTF by polyclonal antibody (100 µg/ml and more) significantly decreased the number of astrocytes (D), but did not affect the numbers of neurons (B) or oligodendrocytes (F). * Significant difference compared to the control (p<0.05, Tukey *post hoc* test).

### Neutralization of endogenous CNTF inhibited the astrocytic differentiation of spinal progenitor cells

To examine the role of endogenous CNTF in differentiation of spinal progenitor cells, we studied the effect of neutralization of endogenous CNTF on the fate of differentiating spinal progenitor cells. Different concentrations (0–500 µg/ml) of anti-CNTF antibody were applied to the differentiation medium at the time of plating. Neutralization of endogenous CNTF by polyclonal antibody (100 µg/ml and more) significantly decreased the number of astrocytes to approximately 20% of the total number of cells compared to 52% in control conditions (100 µg/ml normal rat IgG) (Fig. 4*C, D*). In contrast, neutralization of endogenous CNTF did not change the number of neurons or oligodendrocytes (Fig. 4*A, B, E, F*). There are two possible explanations for these findings. One is that neutralization of endogenous CNTF had an effect on the survival or proliferation of progenitor cells. Alternatively, neutralization of CNTF inhibited the differentiation of progenitor cells into astrocytes.

### Neutralization of endogenous CNTF did not affect the survival or proliferation of spinal progenitor cells

**Figure 5:**
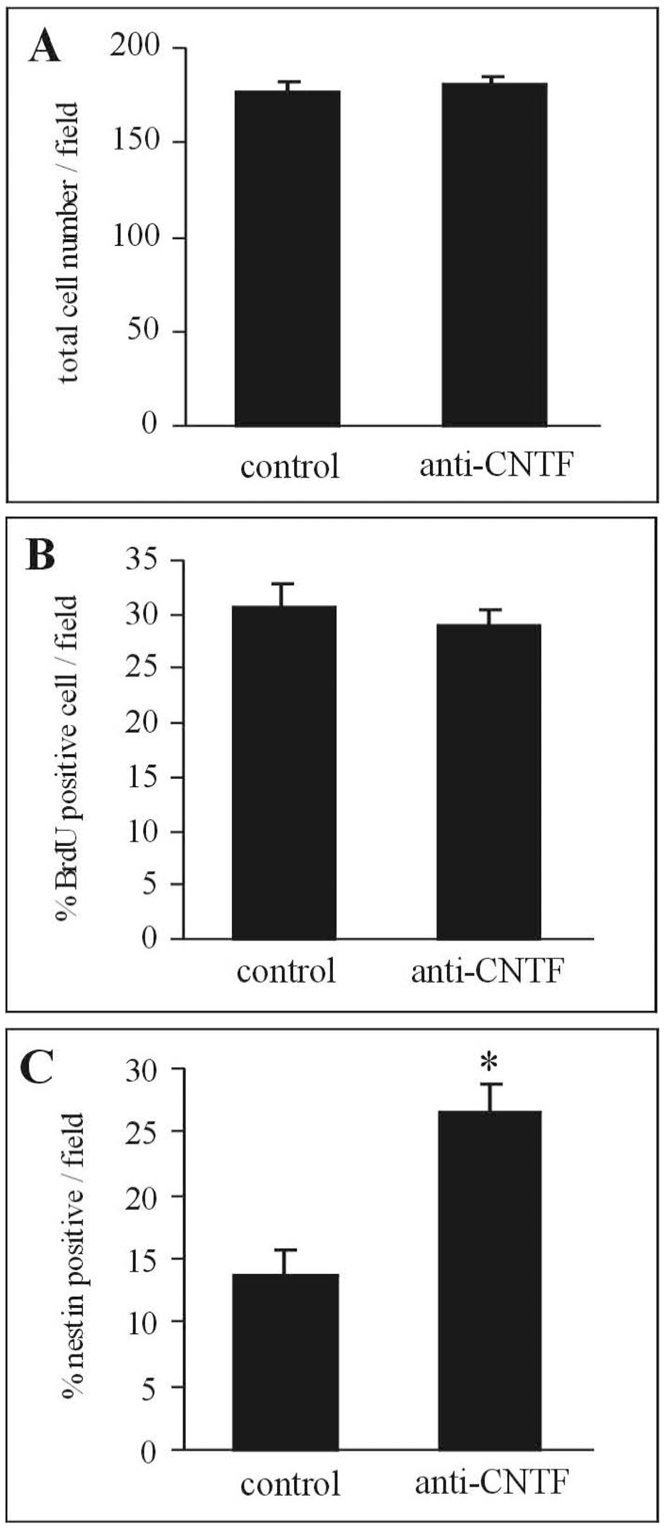
Effect of neutralization of endogenous CNTF on the survival and proliferation of spinal progenitor cells. Comparisons of total cell number (A) and the percentage of BrdU (B) and nestin (C) positive cells to total cell number between control and neutralization groups (anti-CNTF antibody, 100 µg/ml). Neutralization of endogenous CNTF did not affect the survival or proliferation of spinal progenitor cells, but increased the number of undifferentiated progenitor cells expressing nestin (C). * Significant difference between two groups (p<0.05, Mann-Whitney U-test).

To determine the effect of neutralization of endogenous CNTF on survival and proliferation of spinal progenitor cells, we examined the number of total cells and the percentage of nestin-positive or BrdU-positive cell number to the total cell number. Since the application of 100 µg/ml anti-CNTF antibody significantly decreased the number of astrocytes as mentioned above, the subsequent experiments were performed for neutralization of endogenous CNTF using administration of 100 µg/ml anti-CNTF antibody. There was no significant difference in the total cell numbers between control and neutralization groups at 7 div (Fig. 5*A*). Moreover, there were no significant differences in the percentages of BrdU-positive cells between control and neutralization groups at 7 div (Fig. 5*B*). In contrast, the percentage of nestin-positive cells of the neutralization group was significantly higher than that of control group at 7 div (p<0.05) (Fig. 5*C*). These findings suggest that neutralization of endogenous CNTF did not influence the survival or proliferation of the progenitor cells, but rather inhibited the astrocytic differentiation of these cells, resulting in the increase of the number of nestin-positive progenitor cells.

### Neutralization of endogenous CNTF and administration of exogenous BDNF/NT-3 promote the differentiation into neurons/oligodendrocytes

Neutralization of endogenous CNTF inhibited the differentiation of progenitor cells into astrocytes, but did not affect the differentiation into neurons or oligodendrocytes, resulting in the increase of the undifferentiated progenitor cells. These progenitor cells may require additional environmental cues for their differentiation into neurons or oligodendrocytes. Spinal progenitor cells show two major differences in their neurotrophic factor gene expression profile during their differentiation compared to that of the normal spinal cord during embryonic development: 1) there is a down-regulation of endogenous BDNF gene expression and 2) there is a lack of endogenous NT-3 gene expression in the spinal progenitor cells. In order to mimic the neurotrophic factor profile of spinal cord during embryonic development, we applied exogenous BDNF or NT-3 to culture cells in combination with anti-CNTF polyclonal antibody.

**Figure 6:**
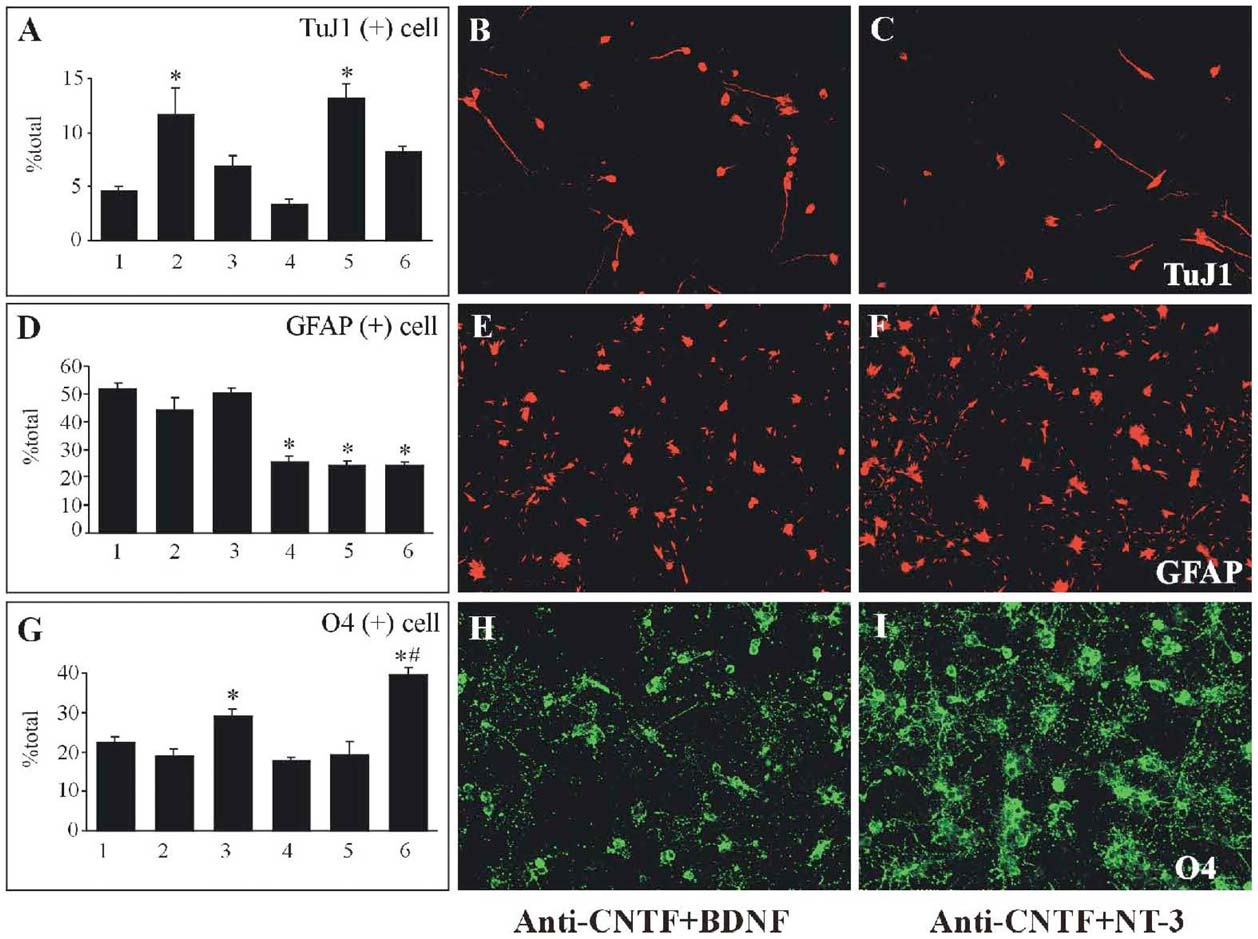
Effects of additional application of exogenous neurotrophic factors on the differentiation of spinal progenitor cells. Treatments of cultures were as follows in each bar graph: (1) control (normal rat IgG 100 µg/ml); (2) BDNF (20 ng/ml); (3) NT-3 (20 ng/ml); (4) anti-CNTF (100 µg/ml); (5) anti-CNTF +BDNF; (6) anti-CNTF + NT-3. At 7 div after each treatment, cells were triple-stained with TuJ1 for neuron, GFAP for astrocyte and O4 for oligodendrocyte. Combined treatments of anti-CNTF plus exogenous BDNF (B, E, H) or NT-3 (C, F, I) not only inhibited the astrocytic differentiation (lanes 5 and 6 in D, E, F), but also promoted the differentiation of spinal progenitor cells into neurons (lane 5 in A, B) or oligodendrocytes (lane 6 in G, I), respectively. The application of exogenous BDNF alone (lane 2 in A) or NT-3 alone (lane 3 in G) also increased the numbers of neuron or oligodendrocytes, respectively. However, the differentiation of progenitor cells into astrocytes was not inhibited by these treatments (lanes 2 and 3 in D). Compared to the cultures treated by NT-3 alone (lane 3 in G), there was a significant increase in the number of oligodendrocytes in the cultures treated by anti-CNTF plus NT-3 (lane 6 in G). * Significant difference compared to the control (lane 1 in A, D, G) (p<0.05, Tukey *post hoc* test). # Significant difference compared to NT-3 alone (lane 3 in G) (p<0.05, Tukey *post hoc* test).

The administration of exogenous BDNF alone increased the number of neurons, but did not change the number of either astrocytes or oligodendrocytes (Fig. 6*A, D, G*). The administration of exogenous NT-3 alone increased the number of oligodendrocytes, but did not change the number of either neurons or astrocytes (Fig. 6*A, D, G*). However, the administration of exogenous BDNF or NT-3 did not have any apparent effect on the survival or proliferation of spinal progenitor cells (data not shown). In combination with anti-CNTF antibody, the administration of exogenous BDNF not only decreased the number of astrocytes (Fig. 6*D, E*) but also increased the number of neurons (Fig. 6*A, B*). There was, however, no significant change in the number of oligodendrocytes (Fig. 6*G, H*). In combination with anti-CNTF antibody, the administration of exogenous NT-3 not only decreased the number of astrocytes (Fig. 6*D, F*) but also increased the number of oligodendrocytes (Fig. 6*G, I*). This treatment did not affect the number of neurons (Fig. 6*A, C*). There was a significant increase in the number of oligodendrocytes in the cultures treated by NT-3 plus anti-CNTF antibody compared to NT-3 alone (Fig. 6*G*).

## Discussion

Multipotent and self-renewing progenitor cells that generate neurons and macroglia are found in most areas of the developing nervous system [1]. Using a neurosphere culture system that allows us to follow the enriched expansion of spinal neural progenitor cells [5], [21], we examined their fate when exposed to conditions that mimic the environment of late embryonic spinal cord in terms of neurotrophic factor profile: neutralization of endogenous CNTF and administration of exogenous BDNF or NT-3. Our results indicate that *endogenous* CNTF plays a role in differentiation of spinal progenitor cells into astrocytes. Furthermore, the additional administration of *exogenous* BDNF or NT-3 promotes their differentiation into neurons or oligodendrocytes, respectively.

### Neurotrophic factor profile of spinal progenitor cells during differentiation is similar to that of spinal cord during postnatal development

Under baseline culture conditions approximately 50% of E14 spinal cord-derived progenitor cells differentiated into astrocytes. This differentiation of spinal progenitor cells towards an astrocytic phenotype may reflect autocrine and/or paracrine interactions between these cells. Such an autocrine mechanism of neurotrophic factor (NT-3 and BDNF) appears to regulate neurogenesis in progenitor cells isolated from the E14 mouse cortex [19], [22]. Thus, endogenous neurotrophic factors may play a role in regulating the differentiation of spinal progenitor cells as well [9]. To the best of our knowledge, this is the first demonstration that spinal progenitor cells express a distinct profile of *endogenous* neurotrophic factor gene expression during their differentiation. We demonstrated that before differentiation, spinal progenitor cells expressed prominent CNTF and BDNF mRNAs in vitro. During their differentiation, *endogenous* BDNF gene expression significantly decreased, whereas CNTF gene expression significantly increased.

This pattern of neurotrophic factor gene expression of spinal progenitor cells in vitro is similar to that of the spinal cord during postnatal development correlated with active gliogenesis, but different from that of the spinal cord during embryonic development correlated with neurogenesis.

In fact, we demonstrated the increase of NGF, BDNF and GDNF gene expression within the developmental spinal cord, which corresponds temporally with the period of differentiation and maturation of neurons [23]–[24], and also the simultaneous down-regulation of NGF, BDNF and GDNF gene expression and up-regulation of CNTF gene expression, which are coincident with postnatal gliogenesis [24], [25].

### Mimicking of the neurotrophic factor profile of spinal cord during embryonic development inhibits the differentiation of spinal progenitor cells into astrocytes, and promotes the differentiation into neurons and oligodendrocytes

Spinal progenitor cells were prepared from E14 embryos, a time when active neurogenesis occurs [23], [24]. Neurotrophic factor gene expression of spinal progenitor cells in vitro differed from that of the embryonic spinal cord during normal development in vivo. The spinal progenitor cells had higher levels of CNTF, and lower levels of BDNF gene expression and failed to express NT-3. We suggest that these differences in the balance of neurotrophic factors environment may promote the differentiation of spinal progenitor cells in vitro into astrocytes. We hypothesize that by altering the neurotrophic factor profile that neural progenitor cells are exposed to in vitro to mimic that of the embryonic spinal cord in situ would inhibit astrocytic differentiation and promote neural differentiation of the spinal progenitor cells.

First, we showed that neutralization of *endogenous* CNTF inhibited the differentiation of spinal progenitor cells into astrocytes, resulting in the increase of the number of undifferentiated progenitor cells that were nestin-positive. Neutralization of endogenous CNTF did not affect the survival or proliferation of spinal progenitor cells. Consistently, previous studies have demonstrated that administration of exogenous CNTF stimulates the two signal-transducing subunits of the CNTFRα-leukemia inhibitory factor receptor β (LIFRβ) and gp130 and induces the differentiation of progenitor cells into astrocytes [11], [15], and that there was a significant reduction in the numbers of neurons and astrocytes within the central nervous system of CNTFRα [26], LIFRβ [27] or gp130 [14] knockout mice. In contrast to mice lacking CNTFRα, LIFRβ or gp130, mice lacking CNTF developed in a normal manner and displayed only mild motor neuron problems later in adulthood without any major neurological abnormalities [28]. Because there is a redundancy of CNTF related neurotrophic factors such as LIF, IL-6 and Oncostatin M in vivo, it is difficult to determine the function of endogenous CNTF by these knockout mice studies. Our in vitro data indicate that *endogenous* CNTF plays a role in inducing the differentiation of spinal progenitor cells into astrocytes, but does not effect their survival or proliferation. A possible explanation of this discrepancy between previous knockout mice studies and our in vitro study is that the redundancy of neurotrophic factors in vivo was not apparent in our cultures because these progenitor cells became more homogenous, at least morphologically, after 2-4 passages.

Although CNTF was previously shown to promote survival of several kinds of neurons such as motor neurons [29], [30], hippocampal neurons [31] and sensory neurons [32] and also survival and maturation of oligodendrocytes [33], [34], neutralization of *endogenous* CNTF did not affect the number of neurons or oligodendrocytes in our cultures. Nakashima et al. demonstrated that in gp130 knockout mice there was a significant neuronal loss at E18.5, but not at E14.5[14]. Their findings suggest that signaling through gp130 is essential for survival of subgroups of differentiated neurons and that this effect depends on the developmental stage of neurons. In our short-term culture system, at least, neutralization of endogenous CNTF did not have apparent effects either on the differentiation of spinal progenitor cells into neurons or oligodendrocytes or on their survival. Further studies using long-term culture system will be needed to determine whether there are any effects of neutralization of endogenous CNTF on their long-term survival and maturation.

Interestingly, while neutralization of *endogenous* CNTF inhibited the differentiation of spinal progenitor cells into astrocytes, this led to an increase in the number of undifferentiated progenitor cells, rather than differentiation into the other cellular phenotypes. This suggests that these cells need additional environmental cues to promote the differentiation of spinal progenitor cells into neurons or oligodendrocytes. Second, we showed that administration of *exogenous* BDNF or NT-3 combined with neutralization of *endogenous* CNTF not only decreased the number of astrocytes, but also increased the number of neurons or oligodendrocytes, respectively. Previously BDNF and NT-3 were shown to promote neuronal differentiation of progenitor cells isolated from E14 striata [4], E14 cortex [19] and E16 hippocampus [18], and CNTF were shown to promote the astrocytic differentiation from NSCs in collaboration with Notch signals [35]. We also demonstrated that treatment of BDNF alone promoted the neuronal differentiation of spinal progenitor cells, but did not affect the glial differentiation. Treatments of BDNF plus anti-CNTF antibody promoted neuronal differentiation and also inhibited the astrocytic differentiation. There was no significant difference in the number of neurons between the cultures treated by BDNF plus anti-CNTF antibody and BDNF alone. Treatment of NT-3 alone promoted the differentiation of spinal progenitor cells into oligodendrocytes, but not into neurons. Treatment of NT-3 plus anti-CNTF antibody promoted the differentiation into oligodendrocytes and inhibited the astrocytic differentiation. Furthermore, there was a significant difference in the number of oligodendrocytes between the cultures treated by NT-3 plus anti-CNTF antibody and NT-3 alone. Rao and colleagues demonstrated that there are heterogeneous populations of E13.5 spinal cord derived progenitor cells including multipotent, neuronal- and glial- restricted progenitor cells [16], [36]. Jean and colleagues reported that in the chemical demyelination model of rat CNS, treatment of NT-3 increased mature oligodendrocyte population and influenced directly on the oligodendrocyte lineage cells to enhance remyelination [37]. A possible explanation of our findings is that neutralization of endogenous CNTF inhibits the differentiation of multipotent and/or glial- restricted progenitor cells into astrocytes, and that they differentiate into oligodendrocytes in the presence of NT-3 and into neurons in the presence of BDNF.

Previous studies demonstrated that there were also endogenous neural stem/progenitor cells in adult spinal cord [21], [38], [39], but after injury these endogenous stem/progenitor cell proliferated [40], migrated to the lesion site and differentiated into astrocytes [41]. These endogenous stem/ progenitor cells may contribute the glial scar formation after spinal cord injury. CNTF gene expression increased significantly, but BDNF and NT-3 gene expression does not increase after spinal cord injury in the adult [42], [43]. This notion is strongly supported by the previous study [44] showing that transplantation of fibroblasts producing BDNF or NT-3 into contused spinal cord promoted the differentiation of endogenous progenitor cells into oligodendrocytes. It was noteworthy that the blocking of CNTF at the beginning of SCI provides a more favorable environment for the differentiation of transplanted NSC and the regeneration of host axons, thereby promoting the functional recovery after SCI [45]. This strategy could be applied to cell transplantation of ES- and iPS-cell derived NSC for SCI.

## Materials and Methods

### Developmental study

Thoracic spinal cord tissues were harvested from Sprague-Dawley rats (Zivic Laboratories, Zelienople, PA) at Embryonic Days 14 (E14) and E19 and Postnatal days 3 (P3), P7 and P17. Following perfusion with ice-cold saline, tissue was frozen on dry-ice quickly and stored at −80 °C until ribonuclease protection assay.

### Spinal Progenitor Cell Culture and Passage

Progenitor cells were cultured using the procedures of Weiss et al (1996b). Thoracic spinal cord tissues were removed from E14 embryos and mechanically dissociated with a fire-polished pipette in culture medium composed of a 1∶1 mixture of DMEM and F-12 nutrient plus 50 U/ml penicillin and 50 mg/ml streptomycin (Life Technologies, Rockville, MD). After dissociation, cells were seeded in culture flasks (Nunclon) at a concentration of 2×10^5^ cells/ml with growth medium. The growth medium contained DMEM and F-12 nutrient (1∶1), 50 U/ml penicillin, 50 mg/ml streptomycin, 0.6% glucose, 2 mM glutamine, 3 mM sodium bicarbonate, 5 mM HEPES buffer, 25 µg/ml insulin, 100 µg/ml transferrin (Life Technologies), 20 nM progesterone, 60 µM putrescine, 30 nM selenium chloride (Sigma, St Louis, MO), and 20 ng/ml EGF and FGF2 (Pepro Tech, Rocky Hill, NJ). After 7 days in vitro (div), cells formed well-developed floating clusters (spheres). To passage spheres, we pelleted 7 div spheres after centrifugation at 400×g for 5 min, resuspended them in fresh medium, and mechanically dissociated them with a fire-polished Pasteur pipette. Single cells were seeded into growth medium in culture flasks at a concentration of 2×10^5^ cells/ml. This procedure resulted in a second generation of spheres.

### Treatments of Cultures

After 2–4 passages, neurospheres were centrifuged and dissociated in EGF/FGF2-free medium. Cells were plated on the polyethyleneimine (PEI)-coated flasks at a density of 2×10^5^ cells/ml in EGF/FGF2-free and 1% fetal bovine serum (FBS) culture medium (differentiation medium). The culture flasks were coated by 0.1% PEI (sodium tetraborate decahyrate 0.15 M, pH 8.5) overnight at room temperature. At 1, 3 and 7 div, these cells were harvested for RNase protection assays of neurotrophic factor gene expression profiles. As a control, cells were also harvested before differentiation following dissociation (0 div).

For immunocytochemistry, after 2–4 passages neurospheres were centrifuged and dissociated in EGF/FGF2-free medium. Cells were plated onto PEI-coated 12 mm coverslips (Fisher Scientific, Pittsburgh, PA) in 24-well plates at a density of 2×10^5^ cells/ml with 1 ml differentiation medium. The role of CNTF in differentiation of spinal progenitor cells was investigated by neutralizing the endogenous CNTF with polyclonal antibody against CNTF IgG. Rat anti-CNTF antibody (Sigma) was applied to the differentiation medium at various concentrations (0–500 µg/ml) at the time of plating. In the control group, we added normal rat IgG (100 µg/ml, Sigma) to the differentiation medium. For analysis of the potential effects of exogenous BDNF or NT-3 on the differentiation of spinal progenitor cells, BDNF or NT-3 (20 ng/ml each, gift from Regeneron Pharmaceuticals, Inc.) was added to the medium at the time of plating and added every 2–3 days. At 7 div, these cultured cells were processed for immunocytochemistry.

### RNase protection assay

Total RNA from each sample was extracted using TRIzol Reagent (Life Technologies) according to the supplier's instructions. RNase protection assays for relative quantification of neurotrophic factor mRNAs [nerve growth factor (NGF), BDNF, NT-3, NT-4, glial-derived neurotrophic factor (GDNF), CNTF] were performed according to the supplier's instructions (PharMingen, San Diego, CA). Briefly, a rat neurotrophin template set (45028P, PharMingen) was labeled with (α-^32^P) uridine triphosphate. 4.6×10^5^ cpm of labeled probe and 5 µg total RNA were used for hybridization. After RNase treatments, the protected probes were resolved on a 5% urea-polyacrylamide gel. Autoradiographs were produced by exposing the labeled gels to Biomax film (Kodak, Rochester, NY) with intensifying screens at −80°C. The gels were exposed to a phosphor screen and analyzed using a phosphoimaging system for quantification (Molecular Dynamics, Sunnyvale, CA). GAPDH was used as an internal control to normalize for loading differences.

### Antibodies

Primary antibodies for indirect immunocytochemistry included a mouse monoclonal antibody to TuJ1 (BAbCO, West Grove, PA), rabbit antiserum to glial fibrillary acidic protein (GFAP, Dako, Carpinteria, CA), mouse-monoclonal antibody to O4 (Boeringer Mannheim, Indianapolis, IL), mouse anti-nestin monoclonal antibody (Chemicon, Temecula, CA), antibodies for trkB and CNTF receptor α (CNTFRα, Santa Cruz, Santa Cruz, CA). Appropriate secondary antibodies were purchased from Jackson ImmunoResearch (West Grove, PA).

### Immunocytochemistry

Immunocytochemical procedures were performed on spinal progenitor cells at 7 div using a slight modification of the procedures of Weiss et al (1996b).

In brief, these cells were fixed with 4% paraformaldehyde for 20 min and washed three times successively with phosphate buffered saline (PBS, pH 7.4). For triple labeling of TuJ1, GFAP and O4, cells were incubated with the primary antibodies for TuJ1 and GFAP diluted in PBS containing 5% normal goat serum and 0.01% Triton-X100 at the same time at 4°C overnight. After washing, cells were incubated with the secondary antibodies of TRITC-conjugated anti-mouse IgG and Cy5-conjugated anti-rabbit IgG at 37°C for 30 min. After washing, cells were incubated with the primary antibody for O4 diluted in PBS containing 5% normal goat serum and 0.01% Triton-X100 at 37°C for 2 hr. After washing, cells were incubated with the secondary antibody of FITC-conjugated anti-mouse IgM at 37°C for 30 min. After washing, the cells were mounted with Fluorsave (Calbiochem, La Jolla, CA). The numbers of immuno-reactive cells and total cells per each field were counted over 20× fields (four coverslips, six random fields per each coverslip) after capturing each image by a confocal laser-scanning microscope (Fluoview, Olympus, Melville, NY). Each immunoreactive cell was counted only when the nucleus was detectable on the 20× field investigated.

### Proliferation Assay

To determine the effect of neutralization of endogenous CNTF on the proliferation of progenitor cells, BrdU labeling of dividing cells was performed as described previously by Ahmed et al. (1995). BrdU (1 µM; Sigma) was added to each of the wells containing progenitor cells (2×10^5^ cells/ml) in 1 ml differentiation medium with or without anti-CNTF antibody (100 µg/ml) at the time of plating for 7 div. The presence of BrdU did not modify total cell number. Cells were stained with ZYMED BrdU staining kit (Zymed, South San Francisco, CA) and counter stained with hematoxylin according to the supplier's instructions. The percentages of BrdU positive cells to total cells of the examined field (four coverslips, six random fields per each coverslip) were calculated.

### Statistics

Each value of RNase protection assays represented mean ± SEM of percentage of each neurotrophic factor mRNA expression to GAPDH obtained from three independent experiments. Statistical comparisons were made using one-way ANOVA with a Tukey *post hoc* test on each neurotrophic factor among different time points. Each value of quantitative analysis of the immunocytochemistry represented mean ± SEM of percentage of immunopositive cell number to total cell number obtained from three independent experiments. Statistical comparisons were made using one-way ANOVA with a Tukey *post hoc* test or Mann-Whitney U-test among different treatment groups. Statistical significance was set at p<0.05.
